# AIE study: a stepping stone to aggregate science

**DOI:** 10.1093/nsr/nwab079

**Published:** 2021-04-30

**Authors:** Zheng Zhao, Ben Zhong Tang

**Affiliations:** Shenzhen Institute of Molecular Aggregate Science and Technology, School of Science and Engineering, The Chinese University of Hong Kong, China; Shenzhen Institute of Molecular Aggregate Science and Technology, School of Science and Engineering, The Chinese University of Hong Kong, China; Department of Chemistry, Hong Kong Branch of Chinese National Engineering Research Center for Tissue Restoration and Reconstruction, The Hong Kong University of Science and Technology, China; Center for Aggregation-Induced Emission, South China University of Technology, China; AIE Institute, China

A molecule, according to Merriam-Webster, is ‘the smallest particle of a substance that retains all the properties of the substance’. This definition laid the foundation for molecular science. Against this reductionist epistemology, holism argues that the whole (e.g. a substance) contains properties that cannot be discovered or understood through the analysis of its parts (e.g. molecules). Aggregation-induced emission (AIE) is an archetypal example of such holism philosophy. A single molecule of AIE luminogen (AIEgen) does not luminesce but its aggregate can emit efficiently upon excitation. AIE study blazed a trail towards aggregate science at higher structural hierarchy and greater system complexity.

The past few decades have witnessed booming progress in AIE research. Some examples of the remarkable advances in the field of AIE study in recent years are presented in this special topic. Peter J. Stang and Xuzhou Yan *et al.* constructed a large family of AIE-active metallacages through coordination-driven self-assembly of metal-organic building blocks and explored potential applications of the new luminogenic metallacages. Kazuo Tanaka's team synthesized AIEgen complexes based on Group 13 elements and exploited their utilities as advanced photoresponsive materials. Xingjie Liang and co-workers utilized AIE-active nanoliposomes to study cellular transportation mechanisms.

The research article by Qi Wang and Weihong Zhu *et al.* reported their design strategy for amphiphilic AIEgens, which overcame the drawbacks of ‘conventional’ AIEgens in the area of sensing, such as the undesired ‘always-on’ fluorescence in aqueous media and the non-specific aggregate signals in lipophilic organelles. The international collaboration team led by Fan Xia, Shixuan Wang and Yuning Hong presented AIEgen-based red blood cell mimicking nanoparticles as photodynamic therapy and immunotherapy agents for anti-tumor treatment. Ying-Wei Yang *et al.* introduced a supramolecular approach to luminogenic hybrid materials with tunable emission and stimuli responses. Xiaoding Lou and co-workers developed a highly sensitive AIEgen-based telomerase biosensor for imaging cellular expression of telomerase reverse transcriptase mRNA and telomerase activity in various cell cycle phases.

Ben Zhong Tang *et al.* discussed important mechanistic issues for the restriction of intramolecular motion (RIM) processes at the quantum mechanics level. Xin Zhang and co-workers envisioned great potential in AIEgens meeting with protein aggregates. The perspective paper by Wenbo Wu and Bin Liu identified challenging issues in the area of AIE study and proposed potential solutions to address the challenges. Jianping Xie *et al.* summarized the progresses made in the study of AIE-active metallic nanoclusters. The perspective article by Zhen Li's team advocated the emerging aggregate chemistry. The research highlight by Zheng Zhao and Ben Zhong Tang introduced a stereoselective divergent strategy developed by Peter J. Stang and Wanxiang Zhao *et al.* for the facile synthesis of tetraarylethylene derivatives, which offered a smart solution to the thorny structural problems faced by tetraphenylethylene (TPE), the most extensively studied AIEgen.

The phenomenon of AIE suggests that an aggregate cannot be comprehended by a simple extrapolation of the behaviors of its molecular components. Entirely new properties can emerge at aggregate level. Unlike isolated molecules, various kinds of interactions come into play when the same or different molecules are aggregated together. AIE is a stepping stone to aggregology, a new framework of scientific research that studies how various forces and processes operate and interplay in an aggregate system. Since every entity beyond a molecule is in principle an aggregate, the scope of aggregate science is huge and its potential is enormous. The nice AIE study showcased in this special topic is expected to catalyze rapid development in the area of aggregate research.

As guest editors, we are sincerely thankful for the great contributions from all the authors and the helpful assistance from the editorial office of *NSR*. We hope this special topic will attract broad attention among scientists and technologists and inspire innovative exploration of new science at aggregate level.

